# Metabolic Actions of Estrogen Receptor Beta (ERβ) are Mediated by a Negative Cross-Talk with PPARγ

**DOI:** 10.1371/journal.pgen.1000108

**Published:** 2008-06-27

**Authors:** Anna Foryst-Ludwig, Markus Clemenz, Stephan Hohmann, Martin Hartge, Christiane Sprang, Nikolaj Frost, Maxim Krikov, Sanjay Bhanot, Rodrigo Barros, Andrea Morani, Jan-Åke Gustafsson, Thomas Unger, Ulrich Kintscher

**Affiliations:** 1 Center for Cardiovascular Research (CCR), Institute of Pharmacology, Charité-Universitätsmedizin Berlin, Berlin, Germany; 2 ISIS Pharmaceuticals, Carlsbad, California, United States of America; 3 Department of Biosciences and Nutrition, Karolinska Institutet, NOVUM, Huddinge, Sweden; Institut de Génétique et de Biologie Moléculaire et Cellulaire, CNRS/INSERM/Université Louis Pasteur, France

## Abstract

Estrogen receptors (ER) are important regulators of metabolic diseases such as obesity and insulin resistance (IR). While ERα seems to have a protective role in such diseases, the function of ERβ is not clear. To characterize the metabolic function of ERβ, we investigated its molecular interaction with a master regulator of insulin signaling/glucose metabolism, the PPARγ, in vitro and in high-fat diet (HFD)-fed ERβ -/- mice (βERKO) mice. Our in vitro experiments showed that ERβ inhibits ligand-mediated PPARγ-transcriptional activity. That resulted in a blockade of PPARγ-induced adipocytic gene expression and in decreased adipogenesis. Overexpression of nuclear coactivators such as SRC1 and TIF2 prevented the ERβ-mediated inhibition of PPARγ activity. Consistent with the in vitro data, we observed increased PPARγ activity in gonadal fat from HFD-fed βERKO mice. In consonance with enhanced PPARγ activation, HFD-fed βERKO mice showed increased body weight gain and fat mass in the presence of improved insulin sensitivity. To directly demonstrate the role of PPARγ in HFD-fed βERKO mice, PPARγ signaling was disrupted by PPARγ antisense oligonucleotide (ASO). Blockade of adipose PPARγ by ASO reversed the phenotype of βERKO mice with an impairment of insulin sensitization and glucose tolerance. Finally, binding of SRC1 and TIF2 to the PPARγ-regulated adiponectin promoter was enhanced in gonadal fat from βERKO mice indicating that the absence of ERβ in adipose tissue results in exaggerated coactivator binding to a PPARγ target promoter. Collectively, our data provide the first evidence that ERβ-deficiency protects against diet-induced IR and glucose intolerance which involves an augmented PPARγ signaling in adipose tissue. Moreover, our data suggest that the coactivators SRC1 and TIF2 are involved in this interaction. Impairment of insulin and glucose metabolism by ERβ may have significant implications for our understanding of hormone receptor-dependent pathophysiology of metabolic diseases, and may be essential for the development of new ERβ-selective agonists.

## Author Summary

In the present study, we demonstrate for the first time a pro-diabetogenic function of the ERβ. Our experiments indicate that ERβ impairs insulin sensitivity and glucose tolerance in mice challenged with a high fat diet (HFD). Loss of ERβ, studied in ERβ -/- mice (βERKO mice), results in increased body weight gain and fat deposition under HFD-treatment. Conversely, absence of ERβ averted accumulation of triglycerides and preserved regular insulin signaling in liver and skeletal muscle. This observation was associated with improved whole-body insulin sensitivity and glucose tolerance. Increased adipose tissue mass in the presence of improved insulin sensitivity and glucose tolerance is usually observed under chronic stimulation of the nuclear hormone receptor PPARγ. In consonance, we show that activation of PPARγ was markedly induced in gonadal fat from βERKO mice and blockade of adipose PPARγ signaling by antisense oligonucleotide injection reversed the metabolic phenotype. Moreover, our cell culture experiments indicate that ERβ is a negative regulator of ligand-induced PPARγ activity in vitro. Finally, we identify SRC1 and TIF2 as key players in the ERβ-PPARγ interaction. In summary, the present study demonstrates that ERβ impairs insulin and glucose metabolism, which may, at least in part, result from a negative cross-talk with adipose PPARγ.

## Introduction

The estrogen receptors (ERs) are members of the nuclear hormone receptor family (NHR) which act as eukaryotic ligand-dependent transcription factors. ERs are involved in the regulation of embryonic development, homeostasis and reproduction. Two major estrogen receptors, alpha and beta (ERα and ERβ), convey the physiological signaling of estrogens (17β-estradiol, E2) [1]. Additionally, ERs are activated by specific synthetic ligands such as raloxifene, tamoxifen, the ERβ-specific ligand diarylpropionitrile (DPN), and the ERβ-specific agonist propylpyrazole-triol (PPT), which belong to the group of selective estrogen receptor modulators (SERMS) [2]–[4].

The prevalence of metabolic diseases such as obesity, insulin resistance and type 2 diabetes has increased dramatically during the recent ten years [5]. Gender differences in the pathophysiology of obesity and metabolic disorders are well established [6]–[8]. However, the molecular mechanisms of sexual dimorphism in metabolic diseases are largely unknown. In addition, lack of ER activation has been implicated in postmenopausal impairment of glucose and lipid metabolism, resulting in visceral fat distribution, insulin resistance and increased cardiovascular risk after menopause [9]. In this context the investigation of ER-signaling and its role in metabolic disorders has gained increasing attention [4],[8].

To identify the ER subtype involved in the regulation of metabolic disorders, studies have been carried out in ER-deficient mice. ERα-deficient (αERKO) mice have profound insulin resistance and impaired glucose tolerance [10]–[13]. These studies indicate that ERα has a protective role in metabolic disorders by improving insulin sensitivity and glucose tolerance. The metabolic function of ERβ is not clear. ERβ knockout mice (βERKO) have a similar body weight and equal fat distribution in comparison to wild type littermates. Additionally, βERKO and wild-type (wt) mice exhibit similar insulin and lipid levels [14]. However, previous studies in βERKO mice were only carried out under low fat diet, which may have concealed a phenotype relevant for human obesity normally induced by high-energy/fat diet.

The peroxisome proliferator-activated receptor gamma (PPARγ) belongs to the NHR family and is a major regulator of glucose and lipid metabolism by modulating energy homeostasis in adipose tissue, skeletal muscle and liver [15]–[17]. Glitazones or thiazolidinediones (TZDs) are high-affinity PPARγ agonists, and act as insulin sensitizers. TZDs induce adipogenesis and adipose tissue remodeling followed by an improvement of glucose tolerance [18]. The role of PPARγ in the control of glucose homeostasis expands beyond its primary action in adipose tissue, and involves the regulation of adipocytokine production such as adiponectin, leptin, and resistin [19]–[21]. Consistently, reduced PPARγ activity has important metabolic and cardiovascular pathophysiological consequences leading to insulin resistance, diabetes and end organ damage [15].

The molecular mechanisms underlying PPARγ function are similar to those of ER-signaling. In a basal state, PPARγ, similar to ERs, is bound to corepressor proteins such as nuclear receptor corepressor (NCoR) or silencing mediator of retinoic acid and thyroid hormone receptor (SMRT) [22]. After binding within the ligand binding domain (LBD), PPARγ ligands induce its heterodimerization with retinoid x receptor alpha (RXRα), and its subsequent interaction with co-activators like steroid receptor coactivators (SRCs) followed by binding to PPARγ response elements (PPREs) within target gene promoters [23]. Importantly, PPARγ is sharing a similar pool of cofactors with ERβ which provides a platform for mutual interactions between these two NHRs [23],[24].

To study the crosstalk between ERβ and PPARγ, we investigated the regulation of PPARγ-mediated transcriptional activity by ERβ. Our in-vitro experiments in 3T3-L1 preadipocytes showed that ERβ inhibits ligand-mediated PPARγ-transcriptional activity. That resulted in the blockade of PPARγ-induced adipocytic gene expression and in decreased adipogenesis. Overexpression of nuclear coactivators such as steroid receptor coactivator 1 (SRC1) and transcriptional intermediary factor 2 (TIF2) prevented the ERβ-mediated inhibition of PPARγ activity, whereas the presence of vitamin D receptor (VDR)-interacting protein 205 (DRIP205) or PPARγ coactivator-1alpha (PGC1α) had no effect indicating a role for distinct nuclear coactivators for ERβ-PPARγ interaction in-vitro. High fat diet (HFD)-fed βERKO mice showed increased body weight and fat mass. In contrast, triglyceride content in liver and muscle was decreased in βERKO mice, which was associated with a marked improvement of hepatic and muscular insulin signaling. Compared to wt, βERKO mice demonstrated improved systemic insulin sensitivity and glucose tolerance. In consonance with the metabolic phenotype and with the in-vitro data, βERKO mice exhibited augmented PPARγ signaling in adipose tissue corresponding to increased food efficiency and significantly elevated RQ (respiratory quotient). Blockade of adipose PPARγ signaling in βERKO mice by PPARγ antisense oligonucleotide injection resulted in a reversal of the βERKO phenotype including body weight reduction and impairment of insulin sensitivity.

In summary, the present data demonstrate that ERβ impairs insulin and glucose metabolism which may, at least in part, result from a negative cross-talk with adipose PPARγ.

## Results

### ERβ Inhibits PPARγ Activity in a Ligand-Independent Manner

**Figure 1:**
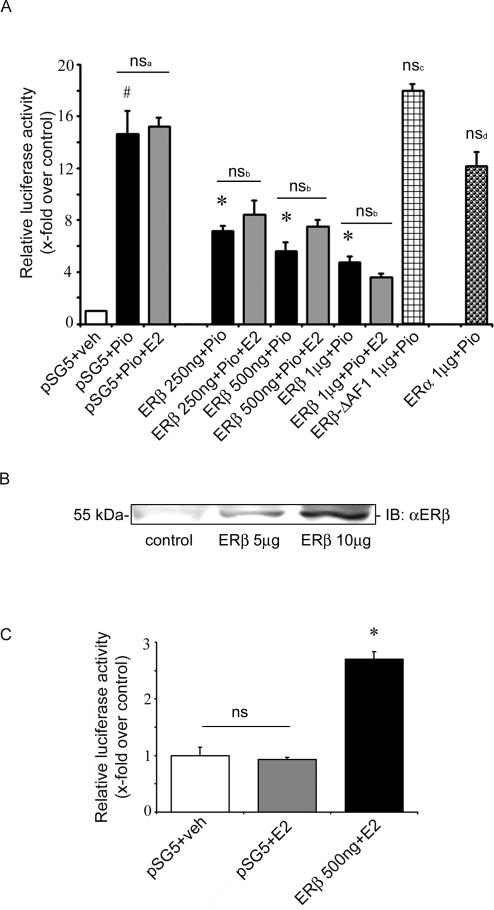
ERβ inhibits PPARγ activity in a ligand-independent manner. A) 3T3-L1 preadipocytes were transfected with the indicated plasmids together with pGal4-hPPARγDEF, pG5TkGL3 and renilla, followed by treatment with 10 µM pioglitazone, 100 nM E2, or in combination as indicated. # p<0.05 vs. pSG5+veh; * p<0.05 vs. pSG5+Pio, ns: not significant vs. pSG5+Pio, ns_a_: not significant vs. ERβ+Pio. B) 3T3-L1 preadipocytes were transfected with ERβ (as indicated), and protein level of ERβ was analysed by Western blot. C) 3T3-L1 preadipocytes were transfected with the indicated plasmids together with pERE-TkGL3, and cells were treated with 100 nM E2, as indicated. * p<0.05 vs. pSG5+E2; ns: not significant vs. pSG5+veh.

In order to demonstrate a molecular interaction between PPARγ and ERβ in a metabolically relevant cell system, we first investigated ligand-dependent PPARγ activity in the presence of ERβ in 3T3-L1 preadipocytes. Cells were treated with the PPARγ-agonist pioglitazone (10 µM), with or without additional E2 stimulation, and PPARγ activation was measured using pGal4-hPPARγDEF/pG5TkGL3 luciferase assay [25]. Upon pioglitazone stimulation, 3T3-L1 preadipocytes showed pronounced PPARγ activation (bar 1+2, Figure 1A). This activation was not affected by co-treatment with ligands for ERβ such as E2 (bar 2 vs. 3, Figure 1A) or DPN (data not shown). Overexpression of ERβ led to a marked inhibition of ligand-dependent PPARγ activity (bar 2 vs. 4+6+8, Figure 1A) which was also corroborated in a PPARγ response element (PPRE) luciferase assay ( Figure S1). This inhibition was E2 (bar 4+6+8 vs. 5+7+9, Figure 1A) and DPN independent (data not shown). The inhibitory effect of ERβ seemed to be isoform specific, since ERα overexpression resulted in no inhibition of PPARγ activity (bar 11, Figure 1A and Figure S2). To further explore the regulation of PPARγ by ERβ, we performed additional experiments coexpressing an activation function 1 domain (AF-1) deleted-ERβ construct in 3T3-L1 cells. Overexpression of this truncated form of ERβ which still contains a functional ligand binding domain (LBD) did not reduce PPARγ activity indicating that ERβ AF-1 is necessary for regulation of PPARγ by ERβ (bar 10, Figure 1A). To assure adequate overexpression and function of ERβ in our system, 3T3-L1 preadipocytes were transiently transfected with ERβ followed by Western blot analysis and transactivation assays using ER response elements (ERE)-luciferase system (Figure 1B, C). Both assays confirmed adequate expression and function of ERβ.

### ERβ Inhibits PPARγ-Dependent Adipocyte Differentiation and Target Gene Expression

**Figure 2:**
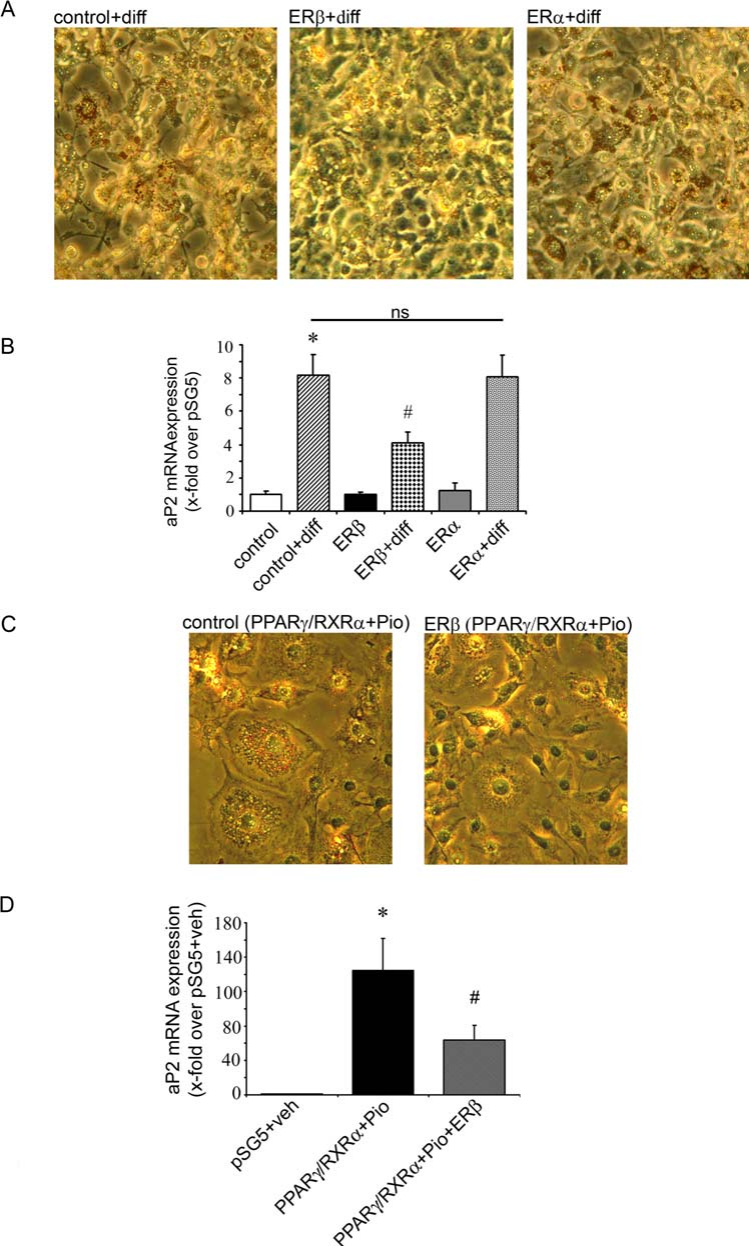
ERβ inhibits PPARγ-dependent adipocyte differentiation and target gene expression. A) 3T3-L1 preadipocytes were transfected with the indicated plasmids and cells were treated with differentiation mix (diff) for 3 days, as indicated. Representative phase-contrast images (20×magnifications) after Oil-Red-O staining are shown. B) 3T3-L1 preadipocytes were transfected with the indicated plasmids and cells were treated with differentiation mix (diff) for 3 days as indicated. mRNA expression of aP2 level is shown, as indicated. Real Time quantitative RT-PCR studies were carried out using total RNA. * p<0.05 vs. control, # p<0,05 vs. control+diff; ns: not significant vs. control+diff. C) 3T3-L1 preadipocytes were transfected with the indicated plasmids and cells were treated with 10 µM pioglitazone for 3 days, as indicated. Representative phase-contrast images (40×magnifications) after Oil-Red-O staining are shown. D) mRNA expression of aP2 levels measured in transfected cells treated with 10 µM pioglitazone for 3 days, as indicated. Real Time quantitative RT-PCR studies were carried out using total RNA. * p<0.05 vs. pSG5+veh, # p<0.05 vs. PPARγ/RXRα+Pio. Values represent means±SEM of at least three independent experiments performed in triplicates.

While our data implicated a negative regulation of ligand-mediated PPARγ transcription by ERβ, we next investigated the regulation of PPARγ-dependent gene expression during 3T3-L1 preadipocyte differentiation. The preadipocytes were transfected with indicated plasmids and differentiated for 3 days using standard differentiation medium [25]. As the full differentiation procedure requires 7-10 days of treatment, the observed effect on fat droplet accumulation and expression pattern are typical for early phase of adipocyte differentiation. The 3T3-L1 cells transfected with ERβ and differentiated for 3 days showed reduced adipogenesis visualized by fat droplet accumulation in comparison to control cells (Figure 2A). Low levels of ERβ could also be detected in untransfected 3T3-L1 cells and its expression was slightly elevated during differentiation (data not shown) underlining the physiological importance of our findings. Overexpression of the ERα isoform in these cells did not show any inhibitory effect on preadipocyte differentiation (Figure 2A). The adipocyte protein 2 (aP2) gene belongs to the classical PPARγ-regulated genes involved in the early phase of adipogenesis [26]. The expression level of aP2 measured by real-time PCR was significantly elevated in the differentiated control cells (bar 2 vs. 1, Figure 2B). Overexpression of ERβ-but not ERα- in these cells led to a significant reduction of aP2 expression (bar 2 vs. 4 and 6, Figure 2B) indicating that endogenous PPARγ activation in 3T3-L1 cells was inhibited by ERβ.

Furthermore pioglitazone (10 µM) treatment of 3T3-L1 cells overexpressing PPARγ/RXRα showed increased adipogenesis, an effect that was markedly inhibited by coexpression of ERβ (Figure 2C). aP2 expression level was also significantly reduced in cells co-expressing ERβ together with PPARγ/RXRα (bar 2 vs. 3, Figure 2D). These data indicate that ERβ inhibits PPARγ-transcriptional activity resulting in the blockade of PPARγ-induced adipocytic target gene expression and amelioration of adipogenesis.

### PPARγ Target Gene Expression and PPARγ Activity Are Increased in βERKO Mice

**Figure 3:**
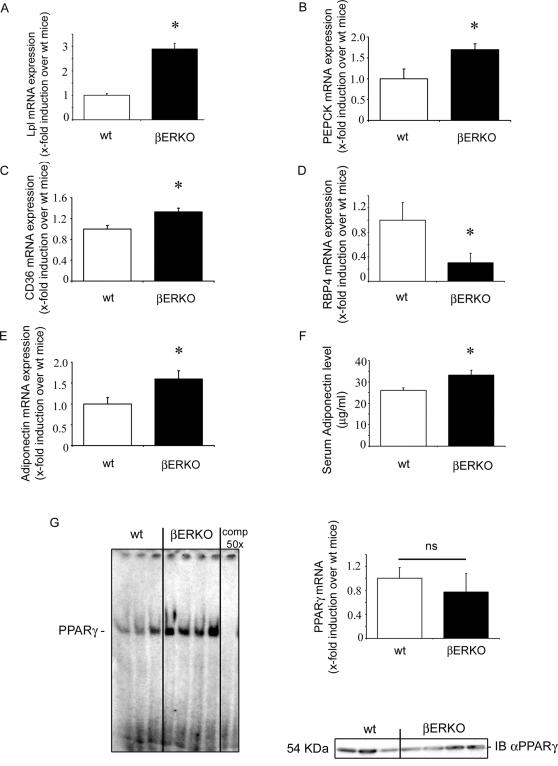
PPARγ target gene expression and PPARγ activity are increased in βERKO mice. A–E) Analysis of Lpl, PEPCK, CD36, RBP4 and adiponectin mRNA expression levels in gonadal fat from HFD-fed wt and βERKO mice. Real-time quantitative RT-PCR studies were carried out using total RNA prepared from gonadal fat isolated from HFD-fed wt and βERKO mice (n = 3 per group). For details, see Materials and Methods and supplemental data. * p<0.05 vs. wt-control. F) Adiponectin levels measured in serum isolated from fasted wt and βERKO mice (n = 10 per group); * p<0.05 vs. wt-control. Values represent means±SEM. G) Nuclear fractions isolated from gonadal fat from HFD-fed wt and βERKO mice (n = 3 and n = 4, respectively) were incubated with ^32^P-labeled PPRE and analyzed by EMSA, as described in Materials and Methods. Real-time quantitative RT-PCR studies for PPARγ mRNA expression in gonadal fat were performed. Additionally 20 µg of the nuclear fraction used in EMSA assay were analyzed in Western Blot using PPARγ-specific antibody.

To investigate ERβ's action on PPARγ in vivo, we studied PPARγ activity and PPARγ target genes in HFD-fed βERKO and wt mice. βERKO mice and their wt littermates were fed HFD containing 60% calories from fat for 12 weeks followed by the analysis of PPARγ-dependent gene expression in gonadal fat tissue. Adipose mRNA expression of PPARγ target genes involved in triglycerides (TG) synthesis such as lipoprotein lipase (Lpl), phosphoenolpyruvate carboxykinase (PEPCK) and CD36 was significantly upregulated in βERKO mice (Figure 3 A–C). Key mediators of insulin and glucose metabolism such as the retinol-binding protein 4 (RBP4) were also regulated in βERKO mice (Figure 3D). Consistently with these findings, adiponectin mRNA expression and adiponectin serum levels were elevated in βERKO mice (Figure 3E, F). No difference of PPARγ target gene regulation between βERKO and wt mice was observed in liver (data not shown).

Positive regulation of a series of adipose PPARγ target genes in βERKO mice suggested a general induction of PPARγ transcription in βERKO mice. To prove this, we performed EMSA assays in gonadal fat from βERKO and wt mice after 12 weeks on HFD. Nuclear fractions isolated from adipose tissues from βERKO mice showed an increased binding/activation of endogenous PPARγ in comparison to wt mice (line 4–7 vs. 1–3, Figure 3G) in the presence of similar PPARγ expression levels, as shown by real-time RT-PCR analysis and Western Blot (Figure 3G). Increased adipose PPARγ target gene expression and PPARγ-DNA binding confirmed an augmented PPARγ signaling in adipose tissue from βERKO mice.

### βERKO Mice Exhibit Enhanced PPARγ Signaling under Pioglitazone Treatment

**Figure 4:**
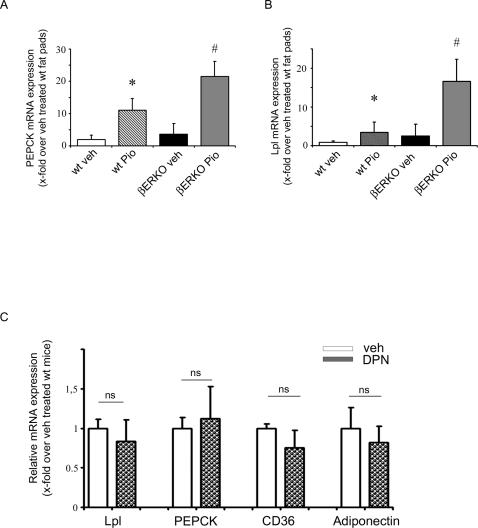
βERKO mice exhibit enhanced PPARγ signaling under pioglitazone treatment. A+B) Explanted gonadal fat pads isolated from wt- and βERKO mice were treated for 24h with 10 µM pioglitazone or vehicle control. Real-time quantitative RT-PCR studies on Lpl and PEPCK expression were carried out using total RNA (n = 4 per group), as indicated. For details, see Materials and Methods and supplemental data. *p<0.05 vs. wt+veh; # p<0.05 vs. wt+Pio. C) ERβ-PPARγ interaction in vivo is ligand independent. Analysis of Lpl, PEPCK, CD36, and adiponectin mRNA expression levels in gonadal fat from soy-free-fed and ovariectomized wt female mice, treated for 21 days with DPN (8 mg/Kg) or vehicle control (n = 4/group). Real-time quantitative RT-PCR studies were carried out using total RNA prepared from gonadal fat. For details, see Materials and Methods and supplemental data. ns: not significant vs. vehicle-treated mice.

To exclude the possibility that the augmented expression of PPARγ target genes measured in HFD-fed βERKO is the result of increased adipose tissue mass, we performed experiments using ex-vivo fat pads isolated from wt and βERKO mice, treated for 24h with 10 µM pioglitazone or vehicle-control, followed by analysis of PPARγ target gene expression using real-time RT-PCR. In this system augmented ligand-induced PPARγ target gene expression mainly results from enhanced PPARγ transcriptional activity and not from increased fat mass. The expression level of PEPCK and Lpl was significantly increased in both wt and βERKO fat pads under pioglitazone treatment (bar 1 vs. 2 and bar 3 vs. 4 Figure 4 A and B). However, pioglitazone-induced PPARγ target gene expression was markedly elevated in βERKO mice compared to wt mice, indicating an augmented PPARγ signaling in the absence of ERβ (bar 2 vs. 4, Figure 4 A and B).

### ERβ-PPARγ Interaction *In Vivo* Is Ligand Independent

To further characterize ERβ ligand dependency for its interaction with PPARγ in the mouse model, additional in-vivo studies were performed in estrogen-depleted, ovariectomized wt mice treated with the ERβ-ligand DPN. Analysis of PPARγ target genes (Lpl, PEPCK, CD36 and adiponectin) in gonadal fat isolated from these mice revealed no significant differences in the expression level between vehicle and DPN-treated rodents indicating ligand independency (Figure 4C). These data are consistent with the in-vitro study in 3T3-L1 preadipocytes, where PPARγ activation was not affected by co-treatment with ligands for ERβ such as E2 (bar 2 vs. 3, Figure 1A) or DPN (data not shown).

### βERKO Mice Exhibit Improved Hepatic and Muscular Insulin Signaling

**Figure 5:**
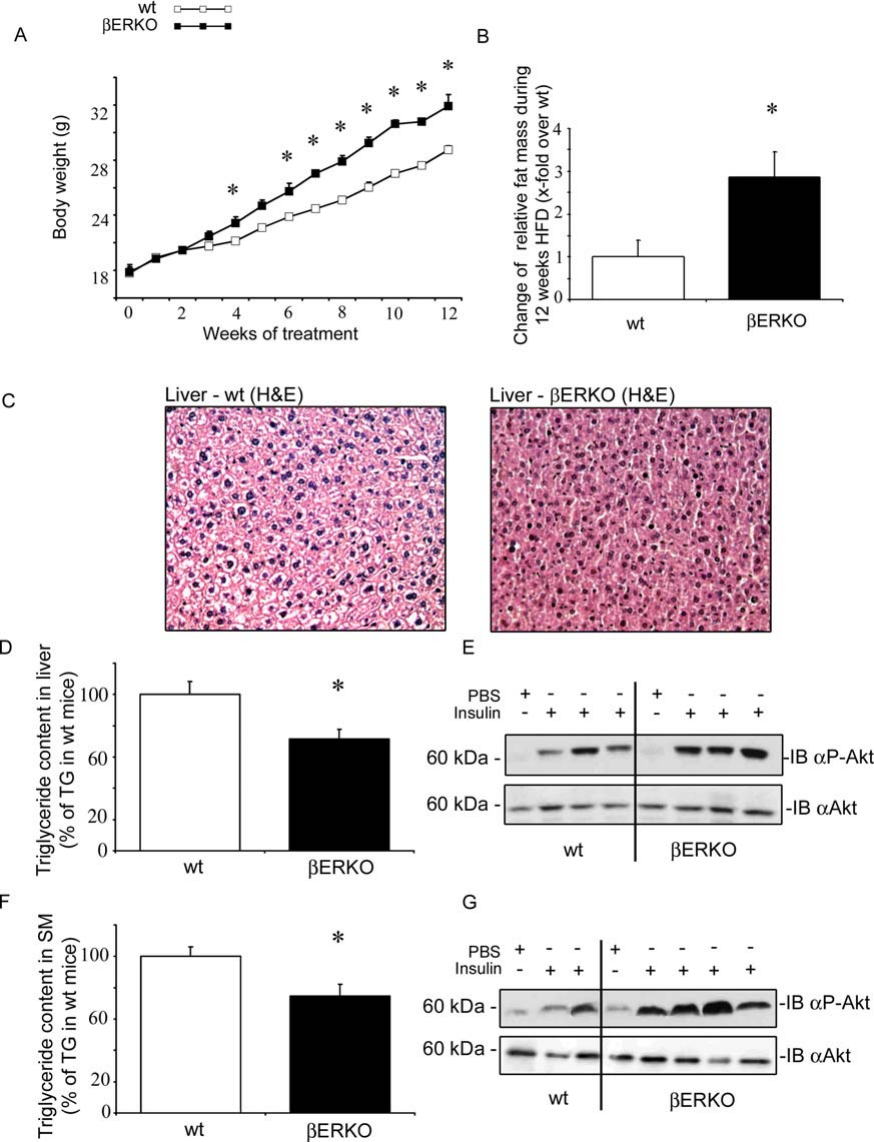
βERKO mice exhibit improved hepatic and muscular insulin signaling. A) Body weight development of HFD-fed wt and βERKO mice. * p<0.05 vs. wt-control. B) Change of relative fat mass during 12 weeks of HFD, presented as x-fold over wt mice. * p<0.05 vs. wt-control C) H&E-stained liver section from HFD-fed wt and βERKO mice. Representative images (20×magnifications) are shown. D+F) Relative TG content in liver and skeletal muscle of HFD-fed wt and βERKO mice (n = 5 per group). * p<0.05 vs. wt-control. E+G) Phosphorylation of Akt kinase in liver and muscle after insulin challenge, as described in Materials and Methods. Representative Western blot analyses using pS473-Akt and total-Akt antibodies are shown.

Given the central role of PPARγ in insulin and glucose metabolism, the metabolic phenotype of βERKO mice was assessed. No difference in fasting/fed blood glucose food intake, and mean arterial blood pressure was observed between βERKO and wt mice under HFD (Table 2). Body weight gain was significantly enhanced in βERKO mice, compared to wt mice (mean BW difference βERKO vs. wt mice after 12 week HFD: 3+/−0.4 g, p<0.05, Figure 5A). Increased body weight in βERKO mice resulted from increased adipose tissue mass. MRI-analysis of body composition demonstrated significantly higher fat mass in βERKO mice compared to wt littermates (Figure 5B), and fat pad weight from gonadal and perirenal depots was increased (Table 1). In contrast, liver weight was significantly reduced in βERKO mice in comparison to wt control littermates (Table 1). Reduced hepatic weight likely resulted from decreased TG-accumulation assessed by H/E-staining of liver tissue sections (Figure 5C), and by TG quantification in dried liver tissue (Figure 5D). In accordance with reduced hepatic TG-content, hepatic insulin signaling was improved. After injection of insulin in the portal vein, liver tissue was dissected and proteins were isolated for Western blot analysis. Insulin-stimulated Akt phosphorylation was enhanced in βERKO mice (Figure 5E and Figure S3). In parallel to decreased TG levels in liver, βERKO mice had decreased muscular TG-accumulation under HFD and improved insulin signaling (Figure 5F, G, and Figure S3). Skeletal muscle and liver are the major insulin responsive tissues, and important sites of glucose metabolism in-vivo. An important mechanism of PPARγ-mediated insulin sensitization involves adipose tissue remodeling and trapping of circulating triglycerides (TG) which protects the liver and skeletal muscle against TG overload. Increased adipose tissue mass in βERKO mice may protect these animals against TG-overload in liver and skeletal muscle resulting in an improvement of hepatic and muscular insulin sensitivity.

**Table 1:** Relative organ weights of HFD-fed βERKO mice.

	wt	βERKO	
**Gonadal Fat** [mg/g BW]	42,88±3,79	60,12±5,54	p<0,05
**Perirenal Fat** [mg/g BW]	11,60±1,41	16,552±1,67	p<0,05
**Liver** [mg/g BW]	42,63±1,2	35,37±1,44	p<0,01
**Heart** [mg/g BW]	5,25±0,24	4,77±0,23	ns

Relative weight of gonadal and perirenal fat, liver and heart (mg/g BW). ns: not significant vs. wt-control (n = 14 per group).

**Table 2:** Metabolic characterization of HFD-fed βERKO mice.

	wt	βERKO	
**Glucose Fed** [mg/dL]	201 ±14,5	196±16,6	ns
**Glucose Fast** [mg/dL]	156±8,54	134±8,287	ns
**Food Intake** [g/day]	2,78±0,06	2,39±0,06	ns
**Blood Pressure** [mean BP mmHg]	88±1,7	90±2,1	ns
**Energy Expenditure** [kcal/kg/h]	2,94±0,32	3,44±0,33	ns
**Locomotor Activity** [counts/h]	2642,4±719	2810,5±784	ns

Glucose level, food intake and mean arterial blood pressure of HFD-fed wt and βERKO mice (n = 14 per group). ns: not significant vs. wt-control. Energy expenditure and locomotor activity was assessed n = 5 mice/ group. Values represent means±SEM.

### Systemic Insulin Sensitivity and Glucose Tolerance Are Improved in βERKO Mice

**Figure 6:**
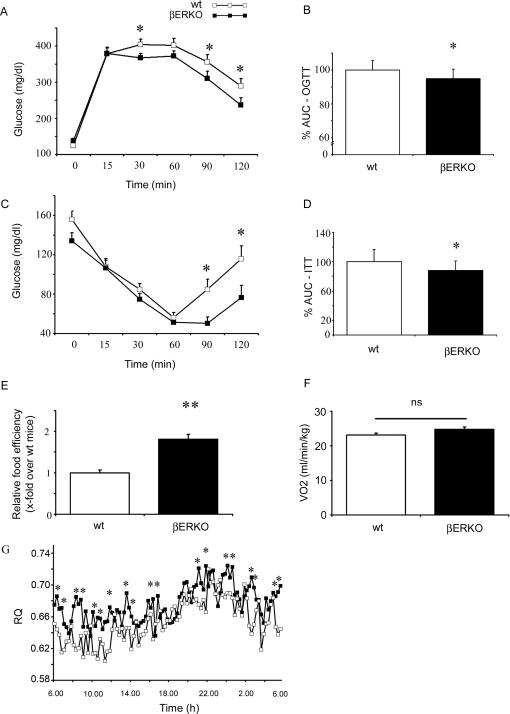
Systemic insulin sensitivity and glucose tolerance are improved in βERKO mice. A+B) Oral glucose tolerance test (OGTT) with 2 g/kg body weight glucose and subsequent glucose analysis from the tail vein was performed as described in Materials and Methods. The area under the curve (AUC) was calculated, as indicated (n = 13 per group). * p<0.05 vs. wt-control. C+D) An Insulin tolerance test (ITT) was performed by an intraperitoneal injection of 0.5 units/kg body weight insulin and glucose analysis from the tail vein, as described in Materials and Methods. The area under the curve (AUC) was calculated, as indicated (n = 13 and n = 10 per group respectively). * p<0.05 vs. wt-control. Values represent means±SEM. E) Food efficiency (ratio of weight gain and food intake) was calculated from change in body weight gain (Figure 1G) and average food intake/ day (Table 2). Data are presented as x-fold over wt mice. ** p<0.01 vs. wt-control. F+G) O_2_ consumption (VO_2_), and respiratory quotient (RQ) from HFD-fed wt and βERKO mice. RQ was calculated as the ratio between CO_2_ produced (VCO_2_) and O_2_ consumed (VO_2_) using the calorimetry system described under methods. * p<0.05 vs. wt-control.

Next we investigated insulin and glucose metabolism in βERKO and wt mice. Whole body glucose disposal was assessed using an oral glucose tolerance test (OGTT) (Figure 6A). Following an oral glucose challenge βERKO mice on HFD had moderately but significantly improved glucose tolerance compared to HFD-fed wt mice (Figure 6A, B). In addition insulin sensitivity measured by an insulin tolerance test (ITT) was improved in comparison to wt mice (Figure 6C, D). No difference in fasting and fed blood glucose was observed between βERKO and wt mice under HFD (Table 2). Despite an increased fat mass in βERKO mice, systemic insulin sensitivity and glucose tolerance were significantly improved under HFD when compared to wt-control. To further examine the enhanced weight gain and fat deposition in βERKO mice, we performed indirect calorimetry and monitored food consumption. Food intake did not differ between wt-control and βERKO mice (Table 2). However, deletion of ERβ resulted in a marked increase of food efficiency (ratio of weight gain and food intake, Figure 6E). No significant difference in O_2_ consumption (Figure 6F), energy expenditure (Table 2), or locomotor activity (Table 2) was detected between βERKO and wt mice. Low RQ values have previously been described for rodents under HFD and in diabetes [27]. Both wt and βERKO mice exhibited low RQ values. βERKO mice had a significantly higher RQ when compared to wt-controls which may be indicative for attenuated fatty acid (FA) oxidation promoting fat accumulation (Figure 6G). These data show that βERKO mice are partially protected against HFD induced insulin resistance. Increased fat mass may likely result from increased food efficiency based on reduced oxidative utilization of fat and increased fat storage. The metabolic phenotype of βERKO mice including increased fat mass, reduced hepatic/muscular TG and improved systemic insulin sensitivity exhibits high similarity to augmented PPARγ activation e.g. under thiazolidinedione (TZD) treatment [28],[29].

### Disruption of PPARγ Signaling by Antisense Oligonucleotide Injection Reversed the Metabolic Phenotype of βERKO Mice

**Figure 7:**
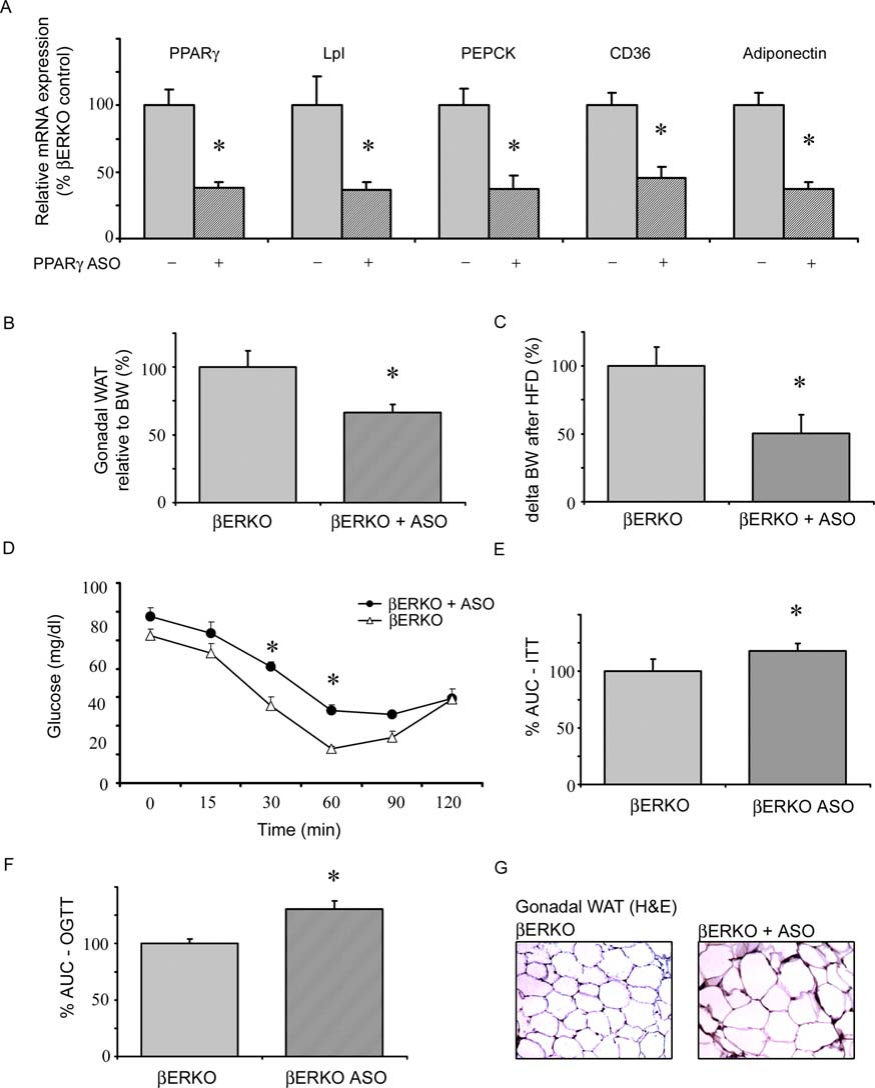
Disruption of PPARγ signaling by antisense oligonucleotide injection reversed the metabolic phenotype of βERKO mice. ASO's complementary to murine PPARγ and control ASO were injected intraperitoneally twice a week into HFD-fed βERKO mice for 6 weeks. A) Analysis of PPARγ, Lpl, PEPCK, CD36, and adiponectin mRNA expression levels in gonadal fat from HFD-fed βERKO mice treated with ASO-control (−) or PPARγ ASO (+). Real-time quantitative RT-PCR studies were carried out using total RNA prepared from gonadal fat. For details, see Materials and Methods and supplemental data. *p<0.05 vs. ASO-control. B+C) Change of relative fat mass and BW during 6 weeks of HFD in βERKO mice treated with ASO-control or PPARγ ASO, presented as % from ASO-control. * p<0.05 vs. ASO-control. D+E) An ITT was performed by an intraperitoneal injection of 0.5 units/kg body weight insulin and glucose analysis from the tail vein, as described in Materials and Methods. The area under the curve (AUC) was calculated, as indicated. * p<0.05 vs. ASO-control. F) An OGTT with 2 g/kg body weight glucose and subsequent glucose analysis from the tail vein was performed as described in Materials and Methods. The area under the curve (AUC) was calculated. * p<0.05 vs. ASO-control. G) H&E-stained gonadal fat section from HFD-fed βERKO mice treated with ASO-control or PPARγ ASO. Representative images (10×magnifications) are shown.

To directly demonstrate the role of PPARγ in HFD-fed βERKO mice, PPARγ signaling was disrupted by intraperitoneal (i.p.) injection of PPARγ antisense oligonucleotide (ASO). HFD-fed βERKO mice were injected twice a week for 6 weeks with either PPARγ ASO or control oligonucleotides. PPARγ expression was significantly reduced in liver of ASO-treated βERKO mice, similar to previously reported results in apoB/BATless mice (data not shown) [30]. However, suppression of hepatic PPARγ by ASO injection is unlikely to play an important role in our model, since hepatic PPARγ signaling did not differ between wt and βERKO mice, respectively. More importantly, i.p. application of PPARγ ASO in βERKO mice resulted in 63±4.8% (p<0.05) reduction of PPARγ expression in gonadal adipose tissue compared to βERKO mice injected with control oligonucleotides (Figure 7A). Accordingly, expression of the PPARγ target genes Lpl, PEPCK, CD36, and adiponectin was markedly decreased in adipose tissue from PPARγ ASO-injected βERKO mice, and adipocyte diameters were increased (Figure 7A, G). These data corroborate a relevant reduction of adipose PPARγ signaling by ASO intervention. Body weight gain and gonadal fat accumulation in HFD-fed-βERKO mice were significantly attenuated by PPARγ-ASO injection (Figure 7B, C). Finally, blockade of adipose PPARγ by ASO led to reversal of the improved insulin response observed in βERKO mice, and to an impairment of insulin sensitivity and glucose tolerance (Figure 7D–F). Together these data underline the importance of adipose PPARγ signaling for the metabolic phenotype observed in βERKO mice.

### ERβ-Mediated Inhibition of PPARγ Activity Involves SRC1 and TIF 2

Nuclear coactivators such as SRC1 and TIF2 are important mediators of ERβ and PPARγ-induced transcriptional activation. It has previously been shown that competition of distinct nuclear receptor (NR) for coactivator binding results in a negative cross-talk between NRs [31]. To prove whether common coactivators are involved in ERβ-PPARγ interactions, SRC1, TIF2, DRIP205 or PGC1α were co-expressed together with ERβ and ligand induced PPARγ activation was measured.

**Figure 8:**
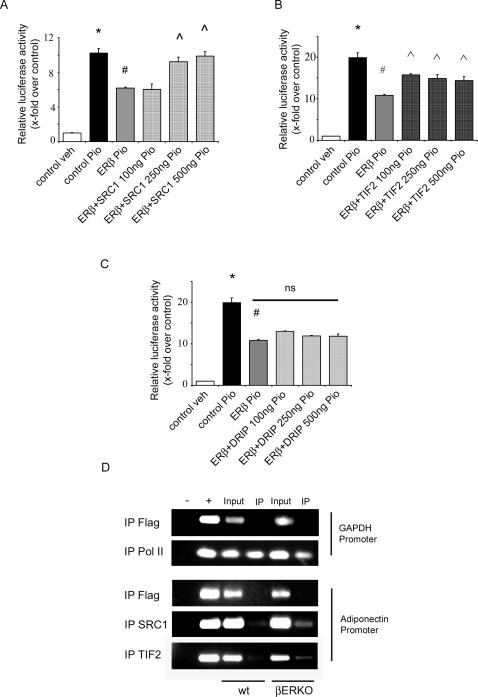
ERβ inhibits PPARγ activity in a ligand-independent manner involving SRC1+TIF2. A–C) 3T3-L1 preadipocytes were transfected with the indicated plasmids together with pGal4-hPPARγDEF, pG5TkGL3 and renilla followed by treatment with 10 µM pioglitazone as indicated. *p<0.05 vs. pSG5+veh; # p<0,05 vs. pSG5+Pio, 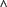 p<0.05 vs. ERβ+Pio, ns: not significant vs. ERβ+Pio. Values represent means±SEM of at least two independent experiments performed in triplicates. D) ChIP experiment with gonadal fat from HFD-fed wt mice and βERKO mice. IP was performed using Flag, RNA Pol II, SRC1 and TIF2 as indicated. (– no template control (NTC), + genomic DNA, input: 1% of the initial probe taken for IP). For details please see Materials and Methods.

Overexpression of SRC1 and TIF2 prevented the ERβ-mediated inhibition of PPARγ activity (Figure 8A, B) whereas the presence of DRIP205 (Figure 8C) and PGC1α (Figure S4) had no effect. To demonstrate that SRC1 and TIF2 are also involved in ERβ-PPARγ interaction in-vivo, we performed ChIP experiments with gonadal fat from HFD-fed βERKO and wt mice. The adiponectin promoter was selected as a PPARγ-target promoter. Binding of SRC1 and TIF2 to the adiponectin promoter was enhanced in gonadal fat from βERKO mice (Figure 8D), indicating that the absence of ERβ in adipose tissue results in exaggerated coactivator binding to a PPARγ target promoter. Together these data suggest that the coactivators SRC1 and TIF2 are involved in the negative regulation of PPARγ by ERβ in vitro and in vivo.

## Discussion

The present study demonstrates that ERβ is a negative regulator of ligand-induced PPARγ activity in-vitro. Consequently, data from βERKO mice suggest that ERβ negatively regulates insulin and glucose metabolism which may, at least in part, result from an impairment of regular adipose tissue function based on a negative cross-talk between ERβ and PPARγ. Loss of ERβ resulted in enhanced body weight gain and fat accumulation in HFD-fed mice. However, absence of ERβ prevented hepatic/ muscular triglyceride overload, preserved regular insulin signaling in liver/ skeletal muscle, and improved whole-body insulin sensitivity and glucose tolerance under HFD. This metabolic phenotype strongly suggested augmented PPARγ signaling in mice lacking ERβ. And indeed, PPARγ target genes and PPARγ-DNA binding were markedly induced in gonadal fat from βERKO mice. Along this line, blockade of adipose PPARγ signaling by PPARγ ASO injection reversed the metabolic changes in βERKO mice.

A mutual signaling cross-talk between ERs and PPARγ has been described previously. PPARγ together with its heterodimeric partner RXRα has been shown to suppress ER-induced target gene expression through competitive binding to an ERE site in the vitellogenin A2 promoter [32]. In accordance with a bidirectional interaction, Wang and colleagues demonstrated that ERs are capable of inhibiting ligand-induced PPARγ activation in two different breast cancer cell lines [33]. In contrast to our results, these authors show that basal and agonist-stimulated PPRE-activity is also blocked by ERα. Transcriptional activity of PPARγ differs markedly depending on the cell system and tissues. The highest level of PPARγ-mediated transcription has been described in adipocytes and adipocytic cell lines, where molecular conditions such as cofactor availability seemed to be optimized [34]. Compared to adipocytes, breast cancer cells exhibit low PPARγ expression and activity reflected by a less than 2-fold induction of PPRE-activity after ligand stimulation [33]. The presence of PPARγ suppression by ERα in breast cancer cells might be a result of weak basal PPARγ transcriptional activity in these cells. In contrast, the pronounced activation of the exogenous PPARγ LBD in 3T3-L1 preadipocytes may require more potent inhibitory stimuli which could not be achieved by ERα overexpression in our system.

Suppression of PPARγ-LBD activation by ERβ did not depend on ERβ ligands which is consistent with previous reports [33]. Also our in vivo studies in estrogen-depleted, ovariectomized wt mice treated with the ERβ-ligand DPN indicate that PPARγ-ERβ interaction is ligand independent. More importantly, overexpression of a truncated form of ERβ containing solely the ERβ-LBD/ AF2 domain did not induce any inhibitory effect on PPARγ suggesting an important role of ERβ's NH_2_-terminal AF1 domain for ERβ-PPARγ interactions. Consistently, activity of the ER-AF1 domain is usually not dependent on ligand activation [35]. Furthermore, Tremblay and coworkers demonstrated that ERβ-AF1 activation involves ligand-independent recruitment of SRC-1, a cofactor involved in ERβ-PPARγ interactions in our study [36]. These data corroborate our observation that PPARγ suppression by ERβ involves the AF1 domain and ligand-independent interactions with the coactivators SRC1 and TIF2. Repression of PPARγ activity through ERβ was reversed by titration of the p160 coactivators, SRC1 and TIF2, suggesting that the suppressive action of ERβ is a result of p160 coactivator interaction with ERβ thereby preventing the binding of PPARγ to the same coactivators. Similar interactions have been described previously for ER interaction with the thyroid receptor [31].

The present study demonstrates for the first time that ERβ impairs insulin sensitivity and glucose tolerance under HFD implicating pro-diabetogenic actions of this receptor. In consonance, we could recently demonstrate that ERβ has a suppressive role on glucose transporter 4 (GLUT4) expression in skeletal muscle [8],[37]. GLUT4 has been identified as the major mediator of insulin-induced glucose uptake in fat and skeletal muscle. In addition, removal of the E2-ERβ signaling by ovariectomy in ERα-deficient mice improved glucose and insulin metabolism supporting the diabetogenic effect of ERβ [12]. Loss of ERβ resulted in a marked augmentation of adipose PPARγ activity in our model indicating that ERβ mediates its metabolic actions by a negative interaction with PPARγ in adipose tissue. This concept is corroborated by a number of observations. HFD-fed βERKO mice exhibited increased adipose tissue mass in the presence of improved insulin sensitivity and glucose tolerance. These metabolic changes are usually observed under chronic PPARγ stimulation [17]. PPARγ has been identified as an essential regulator of whole-body insulin sensitivity. Two major mechanisms have been described: (1) Adipose PPARγ protects non-adipose tissue against excessive lipid overload and maintains normal organ function and insulin responses (liver, skeletal muscle) by preserving regular adipose tissue function, and (2) Adipose PPARγ guarantees a balanced and adequate production of adipocytokine secretion such as adiponectin from adipose tissue, factors which are important mediators of insulin action in peripheral tissues [38]–[40]. Both processes could be observed in βERKO mice. Further support of this notion comes from clinical actions of anti-diabetic PPARγ agonists (TZD) [28],[29]. Activation of PPARγ by TZDs in diabetic patients resembles the phenotype of βERKO mice including improved insulin sensitization and glucose tolerance in the presence of weight gain. We also observed increased food efficiency and changes in nutrient partitioning reflected by an increased RQ in βERKO mice. Loss of ERβ appears to result in attenuated fatty acid (FA) oxidation which may favor the storage of TGs in adipose tissue and increased fat accumulation, and may provide a possible explanation for the enhanced weight gain. Interestingly, treatment of obese mice with a synthetic PPARγ agonist has been shown to mediate similar changes including an increase in food efficiency and higher RQ values [41]. Finally, blockade of PPARγ signaling in adipose tissue of βERKO mice resulted in a reversal of the metabolic phenotype corroborating the importance of adipose PPARγ in the present model. The observed suppression of hepatic PPARγ activity by ASO injection is unlikely to play a major role since the initial metabolic characterization of untreated βERKO mice under HFD did not reveal any dysregulation of hepatic PPARγ signaling. In summary, the metabolic phenotype of βERKO mice is mediated by an augmented adipose PPARγ action, which implies that in the presence of ERβ, PPARγ activity might be partially suppressed.

The notion, that ERβ-PPARγ crosstalk requires receptor-p160 interaction, was underlined by our observations in WAT from βERKO mice. Binding of SRC1 and TIF2 to the PPARγ-regulated adiponectin promoter in WAT was enhanced in the absence of ERβ. It has recently been demonstrated that p160 coactivators are important regulators of PPARγ transcriptional activity in WAT [42]. In particular, TIF2 has been identified as a nuclear coactivator involved in the adipogenic actions of PPARγ. Future experiments are required to define the functional relevance of TIF2 and SRC1 in our model. So far one may conclude that the metabolic phenotype of HFD-fed βERKO mice is, at least in part, explained by increased adipose PPARγ activity as a result of exaggerated binding of p160 coactivators to PPARγ-regulated target gene promoters. Diabetogenic actions of ERβ are of major significance for the pharmaceutical development of new ERβ-selective agonists intended for use against a multitude of diseases such as rheumatoid arthritis or postmenopausal osteoporosis [43],[44]. Despite the high tissue selectivity of such compounds, and despite the fact that the actions observed in our study were ligand-independent, one has to be aware of the potentially deleterious actions of ERβ on insulin- and glucose metabolism. As a precautionary measure metabolic profiling of new ERβ agonist should be performed.

Collectively, our data provide first evidence that ERβ negatively regulates insulin signaling and glucose metabolism that involves an impairment of regular adipose PPARγ function. Moreover our data suggest that the coactivators SRC1 and TIF2 are involved in this inhibition. In consonance, impairment of insulin and glucose metabolism by ERβ has significant implications for our understanding of hormone receptor-dependent pathophysiology of metabolic diseases, and is essential for the development of new ERβ-selective agonists.

## Materials and Methods

### Animal Care and Treatment

Female estrogen receptor β -/- mice (βERKO) received from J.-A. Gustafsson (Karolinska Institutet, Huddinge, Sweden) and their wt littermates were housed in a temperature controlled (25°C) facility with a 12-h light/dark cycle and genotyped using genomic DNA isolation kit (Invitek) and PCR primers described elsewhere [45]. 4–5 week old mice were fed ad libitum with a high-fat diet (60% kcal from fat, [25]) for 12 weeks. Body weight and food intake were determined throughout the experiment. At start and end of treatment, body composition was determined by nuclear magnetic resonance imaging (Bruker's Minispec MQ10). After 12 weeks' treatment, blood samples were collected from overnight-fasted animals by retroorbital venous puncture under isoflurane anesthesia for analysis of serum adiponectin (mouse-adiponectin ELISA; Linco Research) and glucose (colorimetric glucose test; Cypress Diagnostics). An OGTT using a dose of 2 g/kg body weight (BW) glucose and ITT with intraperitoneally injected 0.5 units/kg BW insulin (Actrapid; Novo Nordisk) were performed. Tail vein blood was used for glucose quantification with a glucometer (Precision Xtra; Abbott). Blood pressure was measured invasively in the abdominal aorta using a solid-state pressure transducer catheter (Micro-Tip 3F; Millar Instruments) under isoflurane anesthesia. Afterwards animals were killed and organs were dissected. For immunohistochemical studies organs were fixed in 4% formalin, embedded in paraffin and stained with Haematoxylin/Eosin (H&E); for RNA, Western blot analysis and measurement of TG content isolated organs were frozen in liquid nitrogen; for EMSA and Chromatin IP assays abdominal fat was stored in ice-cold PBS with proteinase inhibitors (Complete Mini, Roche), and immediately proceeded as described below.

For DPN- treatment, 10 week old female C57BL/6J mice were ovariectomized, and after 1 week recovery set on soy-free diet. Subsequently mice were treated for 21 days with DPN (8 mg/kg) or vehicle administered using subcutaneous pellets (Innovative Research of America). Afterwards animals were killed under isoflurane anesthesia and organs were dissected.

All animal procedures were in accordance with institutional guidelines and were approved.

### Antisense Experiments

ASO complementary to murine PPARγ (Gen-BankTM accession number U09138.1), ISIS 141941, 5′-AGTGGTCTTCCATCACGGAG-3′, and ASO control, ISIS 141923, 5′-CCTTCCTGAAGGTTCCTCC-3′ was generously provided by ISIS Pharmaceuticals (Carlsbad, CA, U.S.A.). Both ASO's were injected intraperitoneally twice a week into 6 week-old female βERKO mice (n = 7 per group). Injections were continued over 6 weeks at a dose of 100 mg/kg/week as described previously [30]. At the end of the experiment animals were metabolically phenotyped as described above.

### Energy Expenditure, Locomotor Activity, and RQ

After HFD feeding, βERKO mice and their wt littermates were analyzed for energy expenditure, RQ, and locomotor activity using a custom-made 4-cage calorimetry system (LabMaster; TSE Systems). The instrument consists of a combination of highly sensitive feeding and drinking sensors for automated online measurement. The calorimetry system is an open-circuit system that determines O_2_ consumption, CO_2_ production, and RQ. A photobeam-based activity monitoring system detects and records every ambulatory movement, including rearing and climbing movements, in every cage. All the parameters can be measured continuously. Mice (n = 7 per group) were placed in the calorimetry system cages for 24h.

### Explanted Gonadal Fat Pads Experiments

Tissue samples from gonadal fat were prepared from female wt and βERKO mice. Explanted gonadal fat samples were washed 3 times with ice-cold Hanks Balanced Salt Solution (HBSS) and treated for 24h with 10 µM pioglitazone or vehicle in Dulbecco's modified Eagle's medium F2 (DMEM:F12, Invitrogen). Afterwards tissue samples were washed with ice-cold PBS and RNA extraction was performed using trizol (Invitrogen).

### Cell Culture and Differentiation

3T3-L1 preadipocytes were purchased from the American Type Culture Collection. Preadipocytes were cultured in Dulbecco's modified Eagle's medium with 10% Fetal Bovine Serum (FBS) and 1% Pen-Strep (Invitrogen). For differentiation experiments preadipocytes were grown to confluence and after 12h culture medium was supplemented with methylisobutylxanthine (0.5 mM), dexamethasone (0.25 µM), and insulin (1 µg/ml) in DMEM containing 10% FBS for 72h [25]. Afterwards cells were washed with ice-cold PBS and RNA extraction was performed using trizol (Invitrogen) according to the manufacturer's instructions. For the staining procedure differentiated cells were washed twice with ice-cold PBS, fixed with 4% PFA, and stained for 1h at room temperature with Oil-red-O solution.

### Transfection and Luciferase Reporter Assays

Transient transfection and luciferase assays were performed as previously described [25]. Briefly 3T3-L1 cells were plated in 12-well plates and transfected using lipofectamine 2000 and OptiMEM (Invitrogen) with 100 ng pGal4-hPPARγDEF; 400 ng pG5TkGL3, TIF2-pSG5, DRIP205-pSG5 (kindly provided by B. Staels, Institut Pasteur de Lille, France), 5 ng pRL-CMV, a renilla luciferase reporter vector (Promega), hPPARγ2-pSG5 and hRXRα-pCDNA, pSG5 (Stratagene), hSRC1-pSG5 (kindly provided by M. Parker, Institute of Reproductive and Developmental Biology, Imperial College London, United Kingdom), pERE-TkGL3 (kindly provided by P.J. Kushner, Metabolic Research Unit and Diabetes Center, University of California, San Francisco, USA), hERα-pSG5 and ERβ-pSG5 (kindly provided by P. Chambon, Institut Clinique de la Souris, Illkirch Cedex, France), and PGC1α kindly provided by Addgene, USA. Delta AF1-ERβ-pSG5 was cloned from full length ERβ-pSG5. After 3h of transfection cells were washed, left for 12h in serum deprived medium (0.5% FCS, 1% Pen-Strep), and afterwards treated for 24h with 10 µM pioglitazone (Takeda Pharmaceutical Co. Ltd, Japan) or vehicle (DMSO). When treated with E2 or specific ERβ agonist diarylpropionitrile (DPN), cells were cultivated in phenol red free DMEM and coal-striped FCS. Luciferase activity was measured 36 h after transfection using the dual-luciferase reporter assay system (Promega). Transfection experiments were performed in triplicate and repeated at least three times.

### RNA and Protein Analysis

Total RNA from cultured preadipocytes, abdominal fat tissue and skeletal muscle was isolated using trizol (Invitrogen) according to the manufacturer's instructions. For real-time PCR analysis RNA samples were DNAse digested (Invitrogen), reverse transcribed using Superscript (Promega), RNasin (Promega), dNTPs (Invitrogen), according to the manufacturer's instructions, and used in quantitative PCR reactions in the presence of a fluorescent dye (Sybrgreen, BioRad). Relative abundance of mRNA was calculated after normalization to 18S ribosomal RNA. Primer sequences are provided in Table S1. For Western blot detection of ERβ cells were grown on Φ10 cm plates and transfected with increasing amount of ERβ plasmid or empty vector control. After 24h cells were harvested and WB analysis was performed as following: cells (and tissues for Akt analysis) were lysed in RIPA buffer (50 mM Tris pH 7.5, 150 mM NaCl, 5 mM MgCl_2_, 1% Nonidet P-40, 2.5% glycerol, 1 mM EGTA, 50 mM NaF, 1 mM Na_3_VO_4_, 10 mM Na_4_P_2_O_7_, 100 µM phenylmethylsulfonyl fluoride with proteinase inhibitors (Complete Mini, Roche). Lysates (tissues (30 µg) and cells (20 µg)) were analyzed by immunoblotting using antibody raised against ERβ (H-150, Santa Cruz), antibody raised against pS473- Akt and total-Akt (Cell Signalling), and secondary horseradish-conjugated antibodies (Amersham). For PPARγ immunoblotting, 20 µg of nuclear fractions used for EMSA were analyzed using antibody raised against PPARγ (E-8, Santa Cruz). For detection, enhanced chemiluminescent substrate kit (Amersham) was used.

### EMSA

Nuclear extracts were prepared by using a nonionic detergent method as described previously [46]. The inputs were normalized for protein contents, as ERβ-deficient mice have increased fat tissue mass. Detection of PPARγ was performed with a [^32^P] γATP-labeled PPRE oligo (5′-CAAAACTAGGTCAAAGGTCA-3′ 5′- TGACCTTTGACCTAGTTTTG-3′). The DNA binding reactions were performed with 40 µl of binding buffer (20 µg nuclear extracts, 2 µg of poly(dI-dC), 1 µg of bovine serum albumin (BSA), 5 mM dithiothreitol (DTT), 20 mM HEPES, pH 8.4, 60 mM KCl, and 10% glycerol) for 30 min at 37°C. For competition experiments, a cold oligonucleotide probe was used. The reaction products were analyzed via 5% polyacrylamide gel electrophoresis using 12.5 mM Tris, 12.5 mM boric acid, and 0.25 mM EDTA, pH 8.3. Gels were dried and exposed to Amersham TM film (Amersham Pharmacia Biotech) at −80°C using an intensifying screen.

### Chromatin IP

Abdominal fat tissue (gonadal fat) isolated from wt and βERKO mice was washed in ice-cold PBS with proteinase inhibitors (Complete Mini, Roche), cut into small pieces, and incubated for 12h in 1% formaldehyde, PBS and proteinase inhibitors (Complete Mini, Roche) with rotation at 4°C. Formaldehyde was removed by intensive washing in ice-cold PBS and centrifugation. Samples were lysed in RIPA (with proteinase inhibitors, Complete Mini, Roche), sonicated on ice (Sonopuls HD 2070, 4 times 10s, 100%), and centrifuged. Samples from each group were pooled and protein content of clear phase lysates was measured using a Bradford assay (Amersham). For each immunoprecipitation (IP) 1.5 mg of protein was taken. The volume of the samples was kept constant by using dilution buffer (prepared according to Upstate protocol). For preclearance 90 µl of Protein A Sepharose slurry (Amersham) was added, and the samples were rotated for 1h in 4°C. After centrifugation beads were discarded, and 1% of supernatant volume per aliquot was used as an input control. The residual volume was incubated with 6 µg of appropriate antibodies (anti-Pol II (C-18, Santa Cruz), anti-Flag (Sigma), anti-SRC1 (M-20, Santa Cruz), anti-TIF2 (C-20, Santa Cruz)). The antibody-bound proteins were then precipitated using 300 µl Protein A Sepharose slurry (Amersham), washed and further processed according to the Upstate protocol.

### Quantification of Hepatic/Muscular Triglycerides

Triglyceride-content in skeletal muscle and liver was measured as described previously [47]. Briefly, tissues were homogenized in liquid nitrogen and treated with ice-cold chloroform/methanol/water mixture (2:1:0.8) for 2 min. After centrifugation the aqueous layer was removed and the chloroform layer was decanted. The mixture was incubated at 70°C for chloroform clearance, and the residues were dissolved in isopropanol, and assessed for the triglyceride content using an enzymatic-calorimetric test (Cypress diagnostics) according to the manufacturer's instructions.

### Statistical Analysis

Results from real-time PCR of cell lines, transfections, and animal experiments were analyzed by ANOVA followed by multiple comparison testing or with paired/unpaired t tests, as appropriate. Data are expressed as mean±SEM or as indicated. Results were considered to be statistically significant at p<0.05.

## Supporting Information

Figure S1 ERβ inhibits PPARγ activity in vitro. In order to demonstrate a molecular interaction between PPARγ and ERβ in a metabolically relevant cell system, we first investigated ligand-dependent PPARγ activity in the presence of β in 3T3-L1 preadipocytes. Cells were transfected with 100 ng of PPARγ, 50 ng of RXRα, 700 ng of PPRE-luc, 5 ng of renilla, and increasing amount of ERβ, as indicated. Afterwards cells were treated with the PPARγ-agonist pioglitazone (10 µM), and PPARγ activation was measured using PPRE-luc luciferase assay. Upon pioglitazone stimulation, 3T3-L1 preadipocytes showed increased PPARγ activation. Overexpression of ERβ led to a marked inhibition of ligand-dependent PPARγ activity (bar 1 vs. 2 and 3). # p<0.05 vs. control.
(0.22 MB TIF)

Figure S2 ERα does not inhibit PPARγ activity. 3T3-L1 preadipocytes were transfected with the indicated plasmids together with pGal4-hPPARγDEF, pG5TkGL3 and renilla, followed by treatment with 10 µM pioglitazone or vehicle control; * p<0.05 vs. control+veh; ° p<0.05 vs. control+Pio.
(0.31 MB TIF)

Figure S3 Densitometrical quantification of the Western blot analysis. Densitometrical quantification of the Western blot analysis (Figure 5E/G) was performed by calculating the Akt-P/total Akt ratio. * p<0.05 vs. wt controls.
(0.21 MB TIF)

Figure S4 PGC1α overexpression does not affect ERβ-mediated PPARγ repression. 3T3-L1 preadipocytes were transfected with the PGC1α plasmids together with pGal4-hPPARγDEF, pG5TkGL3 and renilla and 500 ng ERβ followed by treatment with 10 µM pioglitazone as indicated; *p<0.05 vs. pSG5+veh; # p<0,05 vs. pSG5+Pio.
(0.26 MB TIF)

Table S1 Primer sequences used for qRT-PCR and ChIP analysis.
(0.05 MB DOC)
