# Supplementary figures and images for: Metabolic Actions of Estrogen Receptor Beta (ERβ) are Mediated by a Negative Cross-Talk with PPARγ
Source: PLoS Genet. 2008 Jun 27;4(6):e1000108. doi: 10.1371/journal.pgen.1000108 (PMC2432036; doi:10.1371/journal.pgen.1000108)

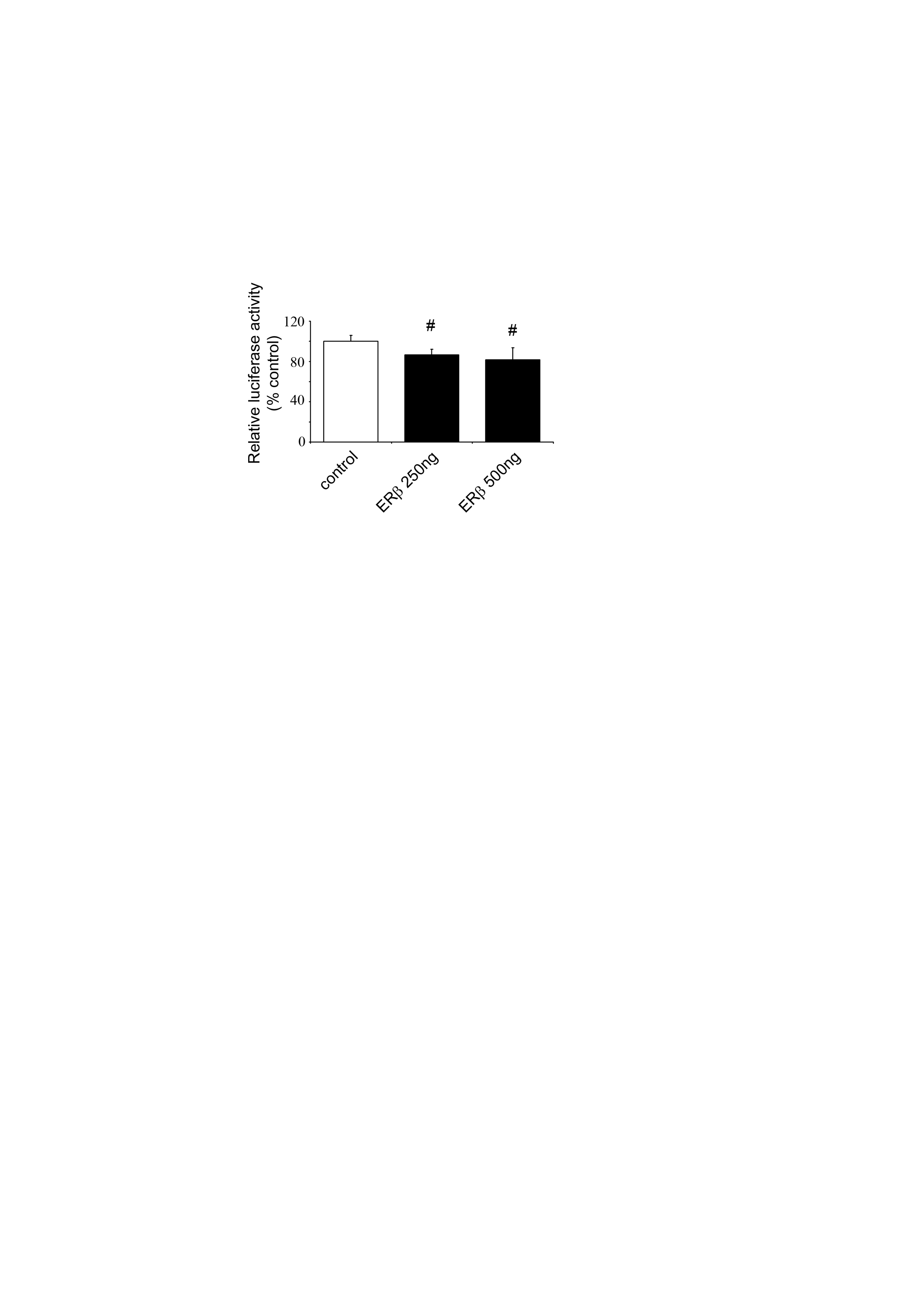

Supplement: Figure S1 — ERβ inhibits PPARγ activity in vitro. In order to demonstrate a molecular interaction between PPARγ and ERβ in a metabolically relevant cell system, we first investigated ligand-dependent PPARγ activity in the presence of β in 3T3-L1 preadipocytes. Cells were transfected with 100 ng of PPARγ, 50 ng of RXRα, 700 ng of PPRE-luc, 5 ng of renilla, and increasing amount of ERβ, as indicated. Afterwards cells were treated with the PPARγ-agonist pioglitazone (10 µM), and PPARγ activation was measured using PPRE-luc luciferase assay. Upon pioglitazone stimulation, 3T3-L1 preadipocytes showed increased PPARγ activation. Overexpression of ERβ led to a marked inhibition of ligand-dependent PPARγ activity (bar 1 vs. 2 and 3). # p<0.05 vs. control. (0.22 MB TIF) [file pgen.1000108.s001.tif]

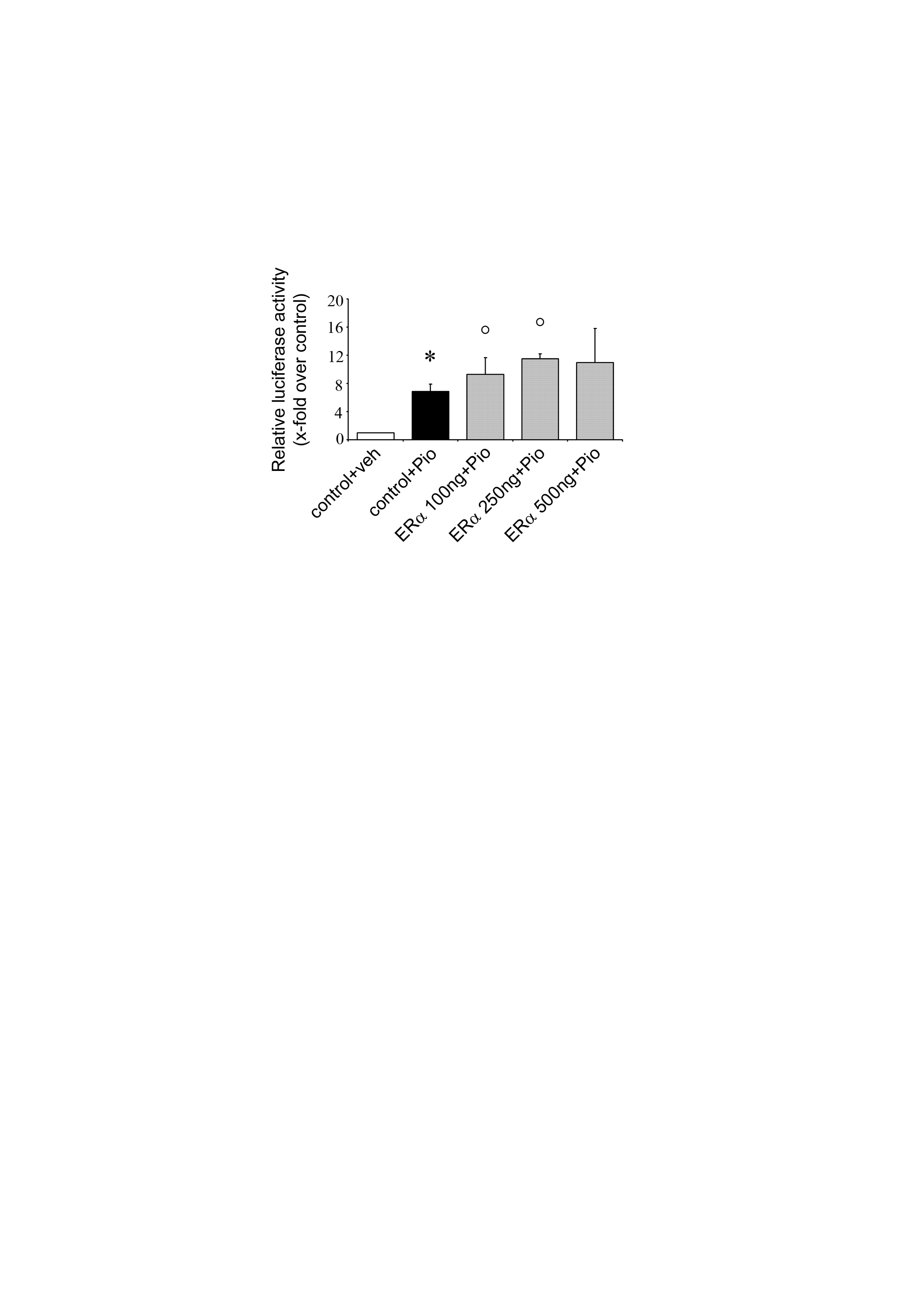

Supplement: Figure S2 — ERα does not inhibit PPARγ activity. 3T3-L1 preadipocytes were transfected with the indicated plasmids together with pGal4-hPPARγDEF, pG5TkGL3 and renilla, followed by treatment with 10 µM pioglitazone or vehicle control; * p<0.05 vs. control+veh; ° p<0.05 vs. control+Pio. (0.31 MB TIF) [file pgen.1000108.s002.tif]

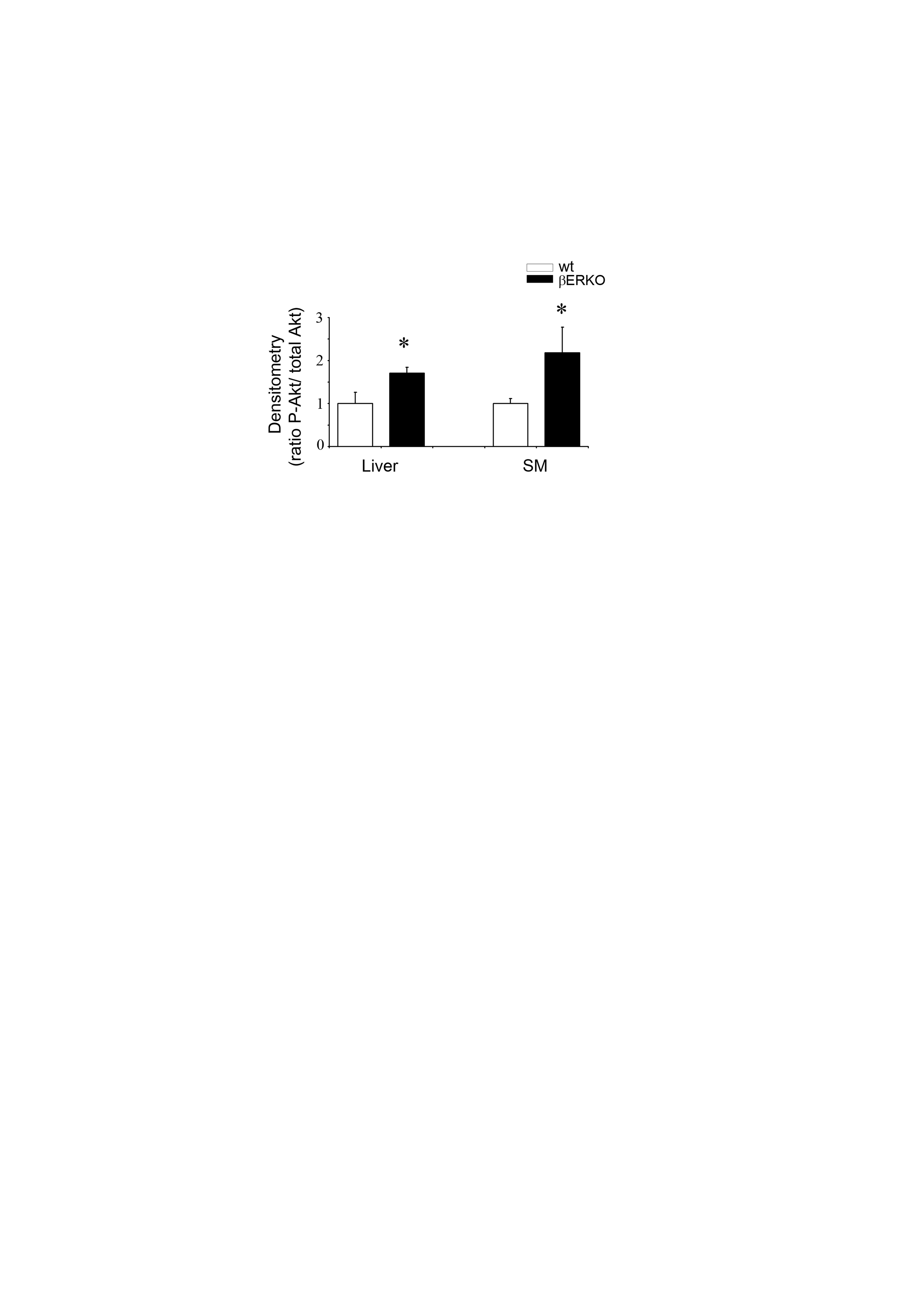

Supplement: Figure S3 — Densitometrical quantification of the Western blot analysis. Densitometrical quantification of the Western blot analysis (Figure 5E/G) was performed by calculating the Akt-P/total Akt ratio. * p<0.05 vs. wt controls. (0.21 MB TIF) [file pgen.1000108.s003.tif]

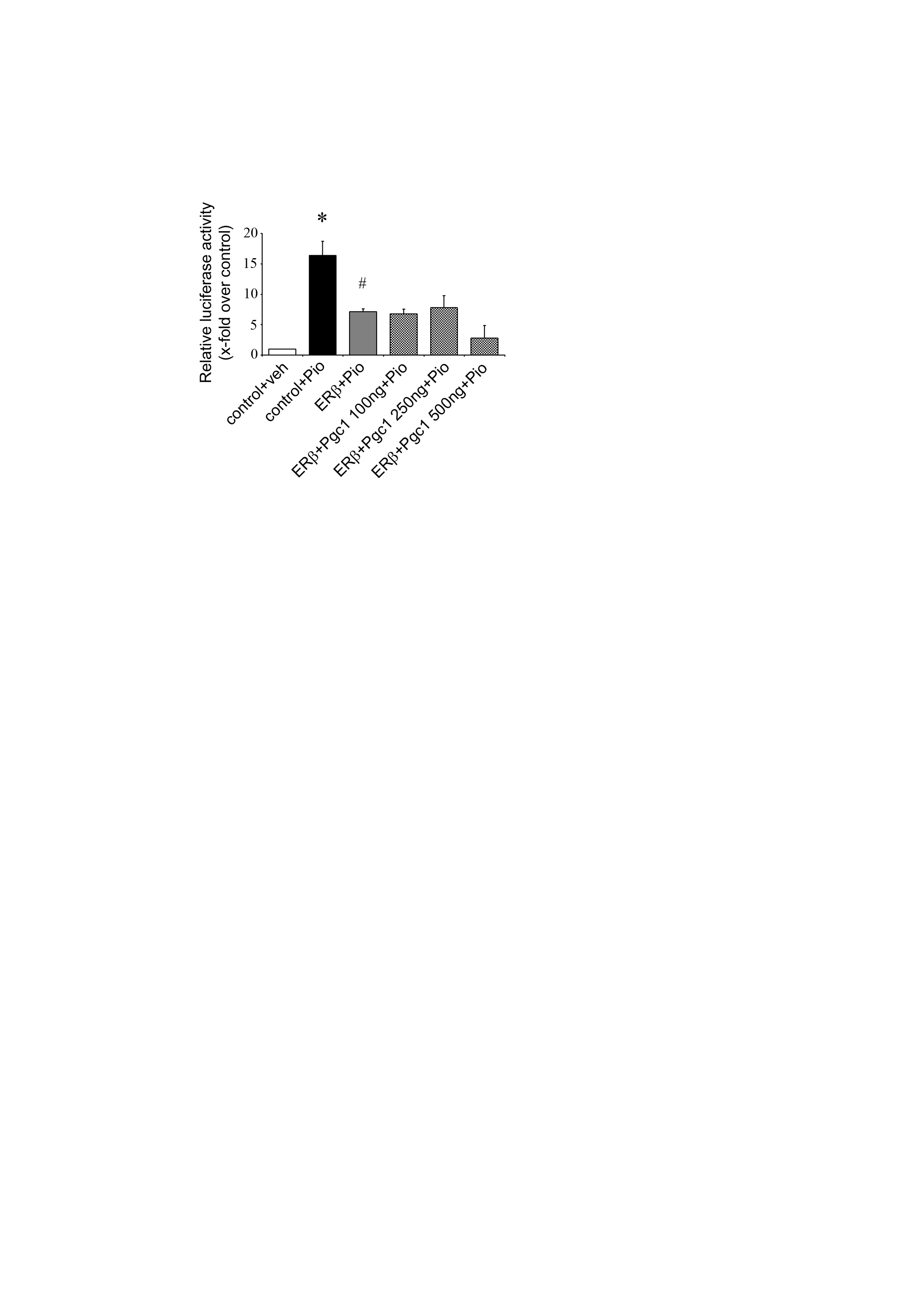

Supplement: Figure S4 — PGC1α overexpression does not affect ERβ-mediated PPARγ repression. 3T3-L1 preadipocytes were transfected with the PGC1α plasmids together with pGal4-hPPARγDEF, pG5TkGL3 and renilla and 500 ng ERβ followed by treatment with 10 µM pioglitazone as indicated; *p<0.05 vs. pSG5+veh; # p<0,05 vs. pSG5+Pio. (0.26 MB TIF) [file pgen.1000108.s004.tif]
