# Supplementary material for: Metabolic Actions of Estrogen Receptor Beta (ERβ) are Mediated by a Negative Cross-Talk with PPARγ
Source: PLoS Genet. 2008 Jun 27;4(6):e1000108. doi: 10.1371/journal.pgen.1000108 (PMC2432036; doi:10.1371/journal.pgen.1000108)
Supplement: Table S1 — Primer sequences used for qRT-PCR and ChIP analysis. (0.05 MB DOC) [file pgen.1000108.s005.doc]

*Table S1. Primer sequences used for qRT-PCR and ChIP analysis.*

| **Gen (mouse)** | **Primers** | **Sequences** |
| --- | --- | --- |
| 18S | Mm 18s for | ACC TGG TTG ATC CTG CCA GTA G |
|  | Mm 18s rev | TTA ATG AGC CAT TCG CAG TTT C |
| ***PPARγ*** | Mm PPARγ for | TTGACAGGAAAGACAACGGA |
|  | Mm PPARγ rev | GAGCAGAGTCACTTGGTCATT |
| ***Lpl*** | Mm lpl for | AGT AGA CTG GTT GTA TCG GG |
|  | Mm lpl rev | AGC GTC ATC AGG AGA AAG G |
| ***AP2*** | Mm aP2 for | TGG AAG ACA GCT CCT CCT CG |
|  | Mm aP2 rev | AAT CCC CAT TTA CGC TGA TGA TC |
| ***ERβ* *genotyping*** | ERβKO exon3 | AGA ATG TTG CAC TGC CCC TGC TGC T |
|  | ERβKO Neo | GCA GCC TCT GTT CCA CAT ACA CTT C |
|  | ERβKO int2 | GGA GTA GAA ACA AGC AAT CCA GAC ATC |
| ***Adiponectin*** | Mm adiponectin for | TCC GGG ACT CTA CTA CTT CTC TTA CCA C |
|  | Mm adiponectin rev | GTC CCC ATC CCC ATA CAC C TG |
| ***CD36*** | Mm CD36 for | gat taa tgg cac aga cgc agc |
|  | Mm CD36 rev | tcc gaa cac agc gta gat ag acc |
| ***RBP4*** | Mm RBP4 for | GCT GTG CCT AGA GAG GCA GTA |
|  | Mm RBP4 rev | TGA AGA CCG GAT GAA AGC TAA |
| ***PEPCK*** | Mm PEPCK for | caa tca cct cct gga aga aca |
|  | Mm PEPCK rev | acc ctc aat ggg tac tcc ttc |
| ***Adiponectin promoter*** | adiponectin promoter for | TGT TGT TGA CTC TCC AGG AC |
|  | adiponectin promoter rev | TAG AGC TTC TGT CAA GCC AT |
| *βActin* | Mm βActin for | gac agg atg cag aag gag att act g |
|  | Mm βActin rev | gct gat cca cat ctg ctg gaa |
| ***ERβ*** | Mm ERβ for | ACT AGT CCA AGC GCC AAG AG |
|  | Mm ERβ rev | AAA GGC CTT ACA TCC TTC ACA |
